# Metropolitan Hospitals, Ancient and Modern

**Published:** 1888-12-15

**Authors:** 


(*To be continued.*)

### Metropolitan Hospitals Ancient and Modern.

### I.—A Retrospect.

It may help in the elucidation of the subject under discussion if we take a retrospective glance at the history of hospital relief in London. Prior to the eighteenth century, with the exception of the two formerly monastic institutions, St. Bartholomew's and St. Thomas's Hospitals, there was absolutely no provision, either legal or voluntary, for the sick poor. Something of the kind was no doubt associated with other religious houses prior to the Reformation, but although a poor-law had existed from the time of Queen Elizabeth, its aim was rather to protect from starvation than to administer to physical ailments. When, in the early part of the last century, public attention was forcibly drawn to the great want, the originators never for a moment supposed that they were interfering with the province of the legislature. On the contrary, they were imbued with the impulse that the duty of succouring the sick and wounded was their prerogative, living and dying in the hope that posterity would maintain and develop those foundations which they had so generously inaugurated.

It is interesting and instructive to note the growth of medical charity in London, which, while it exalts the foresight and benevolence of former generations, in no way lessens a favourable estimate of our own. Of the hundred general and special hospitals situated in, or affiliated with, the metropolis, thirteen date their origin from the last century, the two religious foundations already referred to having become civil establishments about the middle of the sixteenth. Of the others, six were founded during the first quarter of the present century, 18 more were added up to 1850, a large accession amounting to 37 took place between 1850 and 1875, and the remainder have been instituted since then. But it would help better to illustrate the growth of hospital accommodation if we limit our observations to the larger hospitals, which have all undergone more or less extension during the present century. It may be remarked that the population of London at the beginning of the century has been usually estimated at 864,000, by 1841 it had increased to nearly two millions, and at the present time, within the rather more enlarged area of the Registrar-General, it is estimated to contain considerably more than four millions of people. The following table supplies the number of beds in the hospitals having medical schools attached to them, at the three periods mentioned, and a column has been added, showing the average number treated in each during 1887:

| | 1801. Bed Accom. | 1843. Bed Accom. | 1888. Bed Accom. | 1887. Av. No. Occupied. |
|---|---|---|---|---|
| St. Bartholomew's Hospital | 441 | 510 | 650 | 570 |
| St. Thomas's Hospital | 420 | 494 | 600 | 381 |
| Guy's Hospital | 400 | 450 | 600 | 418 |
| The London Hospital | 250 | 330 | 772 | 644 |
| St. George's Hospital | 160 | 320 | 351 | 277 |
| Middlesex Hospital | 80 | 250 | 310 | 176 |
| Westminster Hospital | 80 | 160 | 205 | 172 |
| Charing Cross Hospital | — | 130 | 175 | 125 |
| University College Hospital | — | 130 | 209 | 179 |
| King's College Hospital | — | 120 | 206 | 151 |
| Royal Free Hospital | — | 80 | 160 | 135 |
| St. Mary's Hospital | — | — | 281 | 243 |
| | 1,831 | 2,974 | 4,519 | 3,471 |

In addition to the seven general hospitals which existed in the last century, there were six others of a special character, founded within a few years of each other about the middle of the term; and as their objects have long since been met by other agencies, it is not surprising that they should have remained in a somewhat stationary condition. These comprise the Small-pox Hospital, which, after undergoing several viscissitudes of fortune, has become almost, if not quite, self-supporting; the Lock Hospital, which had more sympathisers with its objects then than now; and the four Lying-in Hospitals previously referred to, whose medical history has not induced posterity to add to the number. If we add to the figures quoted in the table the accommodation provided by other general and by the special hospitals, we have a total of nearly seven thousand beds supported by endowment or by voluntary charity, supplemented by a considerable and increasing amount from patients' payments. This last source of revenue during the past year reached a sum of £41,769, leaving out of the question the monies paid by dispensary patients and by the patients of other institutions which do not participate in the Sunday Fund, but are nevertheless charitable foundations of a curable character, and situated within the metropolitan area. We are constantly hearing of great hospital depression and of the financial difficulties which hospitals have to encounter and surmount, but the evidence gathered collectively from the statistics so often referred to, scarcely justifies the charge of lukewarmness on the part of the people to the wants of our medical charities. The first year's statistics of the Hospital Sunday Fund (1874) place the collective income of the hospitals at £368,305, and their expenditure at £359,487, and that of the convalescent homes at £11,464, with an expenditure of £11,975. In comparison with this, the last year's returns show the hospital income to have increased to £526,653, and that of the convalescent homes to £65,146; the expenditure in the one case amounting to £448,791, exclusive of the investment of legacies, and in the latter to £63,003. To the general income must be added that of the dispensaries, £38,739, and of two endowed hospitals, modestly estimated at £100,000, and we have a rough total of £730,538, without taking into consideration the income of several medical charities and numerous convalescent homes affiliated with the metropolis, which do not participate in the Sunday Fund grants.

There is no evidence here of any falling-off in public support in the maintenance of London hospitals. It may be that from time to time in individual cases the expenditure exceeds the income, and that unless there had been invested capital to draw upon, several well-known hospitals must have long since been in a state of liquidation. But the governing authorities have relied on the hope, by no means delusive, that by the aid of legacies and donations which they are not bound to invest permanently, they may be able in time to surmount their troubles. It would be almost superfluous in the face of these remarks to refer to causes of hospital depression, but there are one or two so often suggested that it would be unfair to pass them over in silence. One of these attributes a failure of interest from the exodus of a large portion of the rich population to distant suburbs, where their charitable proclivities have been diverted into other channels. This is by no means clear. The poor crowd to the environs as well as the rich, and require the aid of the hospitals as much as if they were living within a mile's radius of Charing Cross. A more reasonable cause for lack of sympathy, if it be really wanting, might be traced to the vast improvements which have taken place during the last twenty years in the poor-law medical service of the metropolis. The successive steps taken by the Local Government Board to supplement and, in a measure, to supplant voluntary agencies in the case of the sick are well known. The wretched accommodation provided for them in the workhouses, and the misery inflicted on rich and poor alike by the absence of sufficient means to deal with contagious distempers led to the passing of an act which did more for the sick poor of London than had ever before been attempted by the legislature. By giving the necessary power to local authorities, a series of district infirmaries and asylums for contagious disease was established in and around London, as well as free dispensaries in the most densely-peopled neighbourhoods, while greater facilities were afforded to the voluntary hospitals to rid themselves of infectious diseases which up to that time had greatly embarrassed their sanitary arrangements. There can be little doubt that the great improvement which has taken place in this respect has had a material influence in lessening the strain on the general hospitals, especially in enabling the medical officers to refuse admission to persons suffering from disease of a hopelessly incurable character, and in the better selection of patients for clinical instruction. On the other hand there is no falling-off in the number who apply for relief; for few would be induced to take advantage of the benefits which the workhouse infirmary offers, so long as there was a direct or remote prospect of their being received into the hospital. From an analysis of the accommodation and cost of the infirmaries and sick asylums of the London district, published by the Local Government Board, we learn that they are 24 in number, with a certified accommodation for 11,961 beds, while the district hospitals for infectious disease, including the hospital ships at the mouth of the Thames, have beds for 1,782 patients.

